# Genome-wide analysis reveals distinct global populations of pink bollworm (*Pectinophora gossypiella*)

**DOI:** 10.1038/s41598-023-38504-z

**Published:** 2023-07-20

**Authors:** Paige Matheson, Elahe Parvizi, Jeffrey A. Fabrick, Hamid Anees Siddiqui, Bruce E. Tabashnik, Tom Walsh, Angela McGaughran

**Affiliations:** 1 Te Aka Mātuatua - School of Science, University of Waikato, Hamilton, New Zealand; 2 United States Department of Agriculture Agricultural Research Service, United States Arid Land Agricultural Research Center, Maricopa, AZ 85138 USA; 3 Department of Biology, University of York, York, UK; 4 Department of Entomology, University of Arizona, Tucson, AZ 85721 USA; 5 Commonwealth Scientific Industrial Research Organisation Environment, Clunies Ross St, Acton, ACT 2601 Australia

**Keywords:** Evolutionary biology, Evolutionary genetics

## Abstract

The pink bollworm (*Pectinophora gossypiella*) is one of the world’s most destructive pests of cotton. This invasive lepidopteran occurs in nearly all cotton-growing countries. Its presence in the Ord Valley of North West Australia poses a potential threat to the expanding cotton industry there. To assess this threat and better understand population structure of pink bollworm, we analysed genomic data from individuals collected in the field from North West Australia, India, and Pakistan, as well as from four laboratory colonies that originated in the United States. We identified single nucleotide polymorphisms (SNPs) using a reduced-representation, genotyping-by-sequencing technique (DArTseq). The final filtered dataset included 6355 SNPs and 88 individual genomes that clustered into five groups: Australia, India-Pakistan, and three groups from the United States. We also analysed sequences from Genbank for mitochondrial DNA (mtDNA) locus cytochrome *c* oxidase I (COI) for pink bollworm from six countries. We found low genetic diversity within populations and high differentiation between populations from different continents. The high genetic differentiation between Australia and the other populations and colonies sampled in this study reduces concerns about gene flow to North West Australia, particularly from populations in India and Pakistan that have evolved resistance to transgenic insecticidal cotton. We attribute the observed population structure to pink bollworm’s narrow host plant range and limited dispersal between continents.

## Introduction

**Figure 1:**
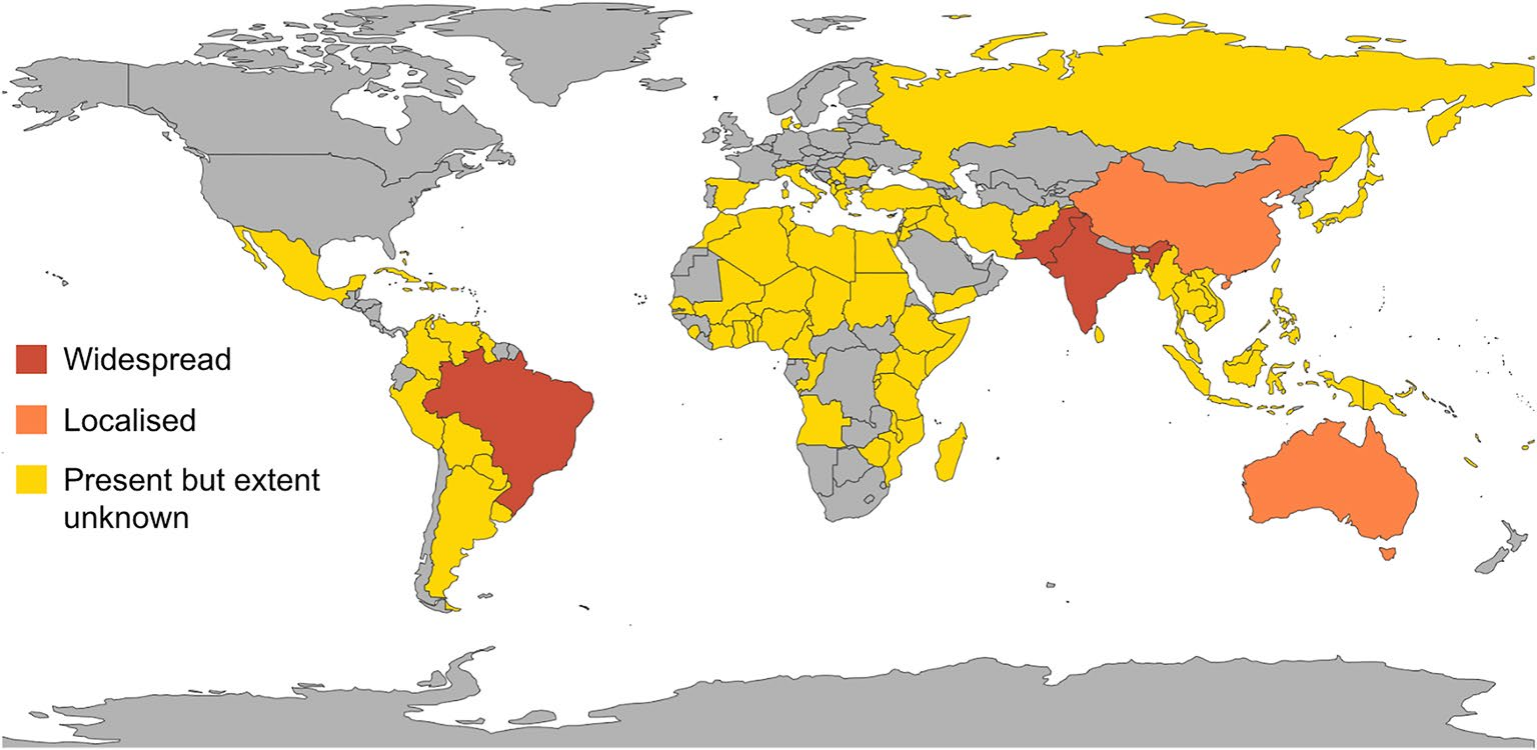
Map depicting where pink bollworm is widespread, localised, and present but extent unknown. Distribution data was extracted from distribution table at https://www.cabidigitallibrary.org/doi/10.1079/cabicompendium.39417 and visualised on a map created in R version 4.1.2^27^ using packages ‘rworldmap’ version 1.3.6^37^ and ‘ggplot2’ version 3.4.2^38^.

Invasive species represent a significant threat to agriculture, due to economic costs associated with management and reduced crop yields^1^. Pink bollworm (*Pectinophora gossypiella*) is a major pest of cotton that has colonised more than 150 countries worldwide, including much of tropical America, Africa, Asia, and Australasia (See Fig. 1)^2,3^. The origin of this invasive lepidopteran pest is not known, but a leading candidate is India^4,5^ where it was first discovered damaging cotton in 1843^6^. It has also been hypothesised to have originated in Australia or South East Asia^7^.

In Australia, pink bollworm was first reported on cotton in Queensland in 1924^8^. Currently, it is thought to occur primarily in Western Australia and the Northern Territory^8–11^, which are over 1000 km from Australia’s primary cotton production areas of New South Wales and southern Queensland. However, pink bollworm’s presence in the Ord Valley in the northern part of Western Australia is a potential threat to the expanding cotton industry there, where over 1000 hectares of cotton were planted in 2020 for the first time since 2011^12^. Cotton genetically engineered to produce insecticidal proteins from the bacterium *Bacillus thuringiensis* (Bt) has been grown in Australia since 1996 and has been effective against pink bollworm in the United States and China^13,14^. However, pink bollworm populations in India and Pakistan have evolved resistance to Bt cotton, with serious practical consequences^15^. In principle, connectivity among populations could result in the introduction of Bt-resistant pink bollworm into North West Australia. Thus, a greater understanding of pink bollworm population structure could be useful for assessing the threat to cotton in Australia, as well for improving surveillance and management elsewhere.

Five previous studies of population structure in pink bollworm have analysed genetic variation based on DNA sequences from one to 13 loci^5–7,16,17^. Two of these studies evaluated only the mitochondrial DNA (mtDNA) cytochrome *c* oxidase subunit I (COI) locus in samples from India^6,17^, two focused primarily on China and used two mtDNA loci ^16^ or 13 microsatellites^5^ respectively, and one was based on a *piggyBac*-like transposon insertion and its flanking sequences^7^. Of these, only the latter included populations from Australia. The conclusions from these studies were mixed, including apparently conflicting results from mtDNA and microsatellite DNA analysis of the same set of populations^5,16^. Population structure of pink bollworm has not been explored previously using genome-wide data that includes Australian samples and a large number of genetic markers. Such analysis may provide increased accuracy and resolution to address these discrepancies.

Here, we aimed to understand pink bollworm population structure by using a genotyping-by-sequencing technique (DArTseq)^18^ to produce genome-wide data in the form of 6355 single nucleotide polymorphisms (SNPs) identified from 88 individual pink bollworm from seven wild populations in Australia, India, and Pakistan; and four laboratory colonies in the U.S. We used these data to assess the population structure of pink bollworm, with a particular focus on the Australian population. We also analysed data from Genbank for mtDNA COI for pink bollworm from six countries. Our results show minimal gene flow between Australia and the other populations that were sampled in this study, which reduces concerns about the threat of introductions of Bt-resistant pink bollworm from India and Pakistan.

## Methods

### Pink bollworm samples

We analysed the genomes of 88 pink bollworm from Australia, India, Pakistan, and the U.S. (Table S1). Male moths were collected from near Kununurra, Western Australia (− 15.65, 128.70) in March and April 2017 using pheromone traps (Agrisense Recharge Lures for *Pectinophora gossypiella*; Entosol (Australia) Pty Ltd., NSW Australia). Fourth instar larvae were collected from fields of Bt cotton producing Cry1Ac + Cry2Ab (Bt toxins) in 2010 from the states of Telangana, Maharashtra, Karnataka, and Andhra Pradesh in India^19^, and from fields of Bt cotton producing Cry1Ac in 2016–2017 from the provinces of Punjab and Sindh in Pakistan.

For pink bollworm from the U.S., we used individuals from four laboratory colonies in Arizona: APHIS-S_1, APHIS-S_2, Bt4R, and Bt4-R2, where this pest was declared eradicated in 2018^20^. Both APHIS-S colonies were obtained from the United States Department of Agriculture (USDA) Animal and Plant Health Inspection Service (APHIS) laboratory in Phoenix, Arizona, where they had been maintained for over 40 years without exposure to Bt toxins or other insecticides^21,22^. Subsets of the APHIS-S colony were provided to the USDA Agricultural Research Service in Maricopa, Arizona in 2006 (APHIS-S_2) and 2018 (APHIS-S_1). Bt4R and Bt4-R2 are laboratory-selected resistant colonies: Bt4R was derived from the Bt-susceptible Western Cotton Research Laboratory (WCRL) colony from 2007 to 2008 by laboratory selection for Cry1Ac resistance^23^. Bt4-R2 was obtained from Bt4R in 2010 and has 28-fold resistance to Cry1Ac and > 10,000-fold resistance to Cry2Ab^24^. Larvae from all four colonies were reared in the laboratory on wheat germ diet^25^ at 26 °C and a photoperiod of 14 h light:10 h dark.

### DArTseq genotyping

We sent 90 pink bollworm samples to Diversity Arrays Technology (DarT Pty Ltd, Canberra, Australia) for DNA extraction, quantification, and genotyping (see Supplementary Information Table S1 for further sample details). DarTseq™ is a restriction enzyme-based, complexity reduction method that employs a next generation sequencing platform, as detailed by Kilian et al. and Georges et al.^18,26^. Short-read sequences were then processed following the DArTseq bioinformatic pipeline to yield genotyping data in the form of SNPs^26^.

### SNP filtering

We received genotyping outputs in the DarT ‘2 row’ format, where alleles are scored “0” for homozygous reference state, “1” for heterozygous, and “2” for homozygous alternate (or SNP) state^26^. This initial dataset contained 59,262 SNPs across 90 individuals. Using R version 4.1.2^27^, we converted this data into a genlight object using ‘adegenet’ version 2.1.7^28^, and then used ‘dartR’ version 2.0.4^29^ and ‘radiator’ version 1.2.2^30^ for data manipulation and filtering. We filtered SNPs by reproducibility (threshold: 0.98), call rate (threshold: 0.95), and minor allele frequency (threshold: 0.02). The final filtered dataset contained 88 individual genotypes, 6355 SNP markers, and 1.41% missing data.

### Heterozygosity and inbreeding

In R, we used ‘hierfstat’ version 0.5.11^31^ to calculate observed (Ho) and expected (He) heterozygosity, as well as inbreeding coefficients (F_IS_; a metric that ranges from − 1 to 1, where values close to 0 meet the neutral expectation, values that approach 1 indicate a deficit of heterozygotes indicating inbreeding, and values approaching − 1 indicate an excess of heterozygotes) for each population from the filtered dataset.

### Population structure

We used R to analyse genetic variation between and within populations by performing an Analysis of Molecular Variance (AMOVA) with the ‘poppr’ package version 2.9.3^32^. We randomly permuted the AMOVA output 1000 times to test if populations and colonies were significantly different using the function ‘randtest’ from the package ‘ade4’ version 1.7.19^33^. We then estimated pairwise genetic differentiation (F_ST_) between populations and colonies using the function ‘genet.dist’ and method ‘WC84’ from the ‘hierfstat’ package, version 0.5.11^34,35^.

We conducted a principal component analysis based on Euclidean genetic distances, using the function ‘glPCA’ implemented in the ‘adegenet’ package in R. We performed two analyses, one with only the 11 populations and colonies of pink bollworm, and another that also included an ‘outgroup’ population of spotted pink bollworm (*Pectinophora scutigera*; see Fig. S1).

We calculated individual admixture coefficients by first converting SNP data into ‘STRUCTURE’ format using the ‘gl2faststructure’ function implemented in the R package ‘dartR’, and then to ‘.geno’ format using the ‘struct2geno’ function from ‘LEA’ version 3.6.0^36^. We ran sparse non-negative matrix factorisation on individuals using the ‘sNMF’ function also implemented in ‘LEA’ and analysed K values of 1–10, with 100 replications for each K value. We identified the K value that best explained our results using the cross-entropy criterion (Fig. S2). Admixture results were presented on a map created using the R packages ‘rworldmap’ version 1.3.6^37^ and ggplot2 version 3.4.2^38^.

To examine relationships among samples in a phylogenetic context, we converted our SNP data to variant call format (VCF) using ‘radiator’ version 1.2.2^30^ and then to phylip format using the command ‘vcf2phylip’ script (available at: https://github.com/edgardomortiz/vcf2phylip). A maximum likelihood (ML) phylogeny was constructed in Iqtree version 1.2.1^39^ using the best-fit substitution model automatically selected by the software, with 10,000 bootstrap iterations to assess clade support. The resulting output was read into R using ‘ape’ version 5.6.2^40^ for data visualisation.

Finally, to further investigate genetic diversity and population structure of global pink bollworm populations, we obtained 31 mitochondrial COI sequences in FASTA format from GenBank (Table S2) for populations from Australia, India, Pakistan, the U.S., Israel, and Kenya. COI sequences were aligned using MEGA version 11^41^. The nucleotide diversity of each population was then calculated using the ‘nuc.div’ function in R from the ‘pegas’ package version 1.1^42^. COI sequences were converted to haplotypes using the same package, and the function ‘haploNet’ was used to construct a haplotype network.

## Results

### Heterozygosity and inbreeding

Observed heterozygosity (Ho) was low within each of the 11 populations and colonies studied (range = 0.060–0.207, Table 1). As expected, mean Ho was lower for the four U.S. laboratory colonies (0.09) than the seven field populations from Australia, India, and Pakistan (0.19) (t_9_ = 4.87; *P* < 0.001). Mean Ho was significantly higher for the field populations from India and Pakistan (0.20) than for Australia (0.14) (t_5_ = 11.5, *P* < 0.001). Ho was not correlated with the number of field sites sampled per population across the seven field populations (r_5_ = 0.34; *P* = 0.46) nor the number of individuals sampled per population across all 11 populations and colonies (r_9_ = 0.12; *P* = 0.72).

**Table 1:** Location, number of field sites and individuals (n) sampled, observed heterozygosity (Ho), expected heterozygosity (He), and inbreeding coefficient (F_IS_) for 11 populations of pink bollworm.

Population	Country	State or province	Field sites	n	Ho	He	F_IS_
Aus-K	Australia	Western Australia	1	12	0.141	0.209	0.280
Ind-A	India	Andhra Pradesh	3	10	0.197	0.231	0.117
Ind-K	India	Karnataka	1	4	0.194	0.233	0.110
Ind-M	India	Maharashtra	3	9	0.195	0.230	0.121
Ind-T	India	Telangana	2	5	0.198	0.232	0.101
Pak-P	Pakistan	Punjab	5	14	0.199	0.236	0.129
Pak-S	Pakistan	Sindh	1	4	0.207	0.235	0.068
APHIS-S_1	U.S.	Arizona	NA	12	0.160	0.178	0.083
APHIS-S_2	U.S.	Arizona	NA	11	0.060	0.060	0.000
Bt4R	U.S.	Arizona	NA	4	0.080	0.089	0.055
Bt4-R2	U.S.	Arizona	NA	3	0.072	0.075	-0.015

Overall, observed heterozygosity (mean = 0.15) was significantly lower than expected heterozygosity (He) (mean = 0.18) (paired *t*-test, t_10_ = 4.72; *P* < 0.001, Table 1). The mean inbreeding coefficient (F_IS_) was higher for field populations (0.13) than laboratory colonies (0.03) (t_9_ = 2.63, *P* = 0.03) (Table 1).

### Population structure

AMOVA indicated that 25.6% of genetic variation was partitioned between populations and colonies, while 12.3 and 62.1% of variation was partitioned between and within individuals, respectively (*P* < 0.001 in all cases; Table 2).

**Table 2:** Analysis of molecular variance (AMOVA) to assess variation between populations, and between and within individuals, for 11 populations of pink bollworm.

Source	df	SS	MS	Est.Var	PV	*P* value
Between populations	10	79,799.7	7979.9	421.4	25.6	< 0.001
Between individuals	77	107,396.5	1394.8	193.2	12.3	< 0.001
Within individuals	88	88,741.7	1008.4	62.1	62.1	< 0.001
Total	175	275,937.9	1576.8	1623.0	100.0	–

*df* degrees of freedom, *SS* sum of squares, *MS* mean square, *Est. Var* estimated variance, *PV* percentage of variance.

**Figure 2:**
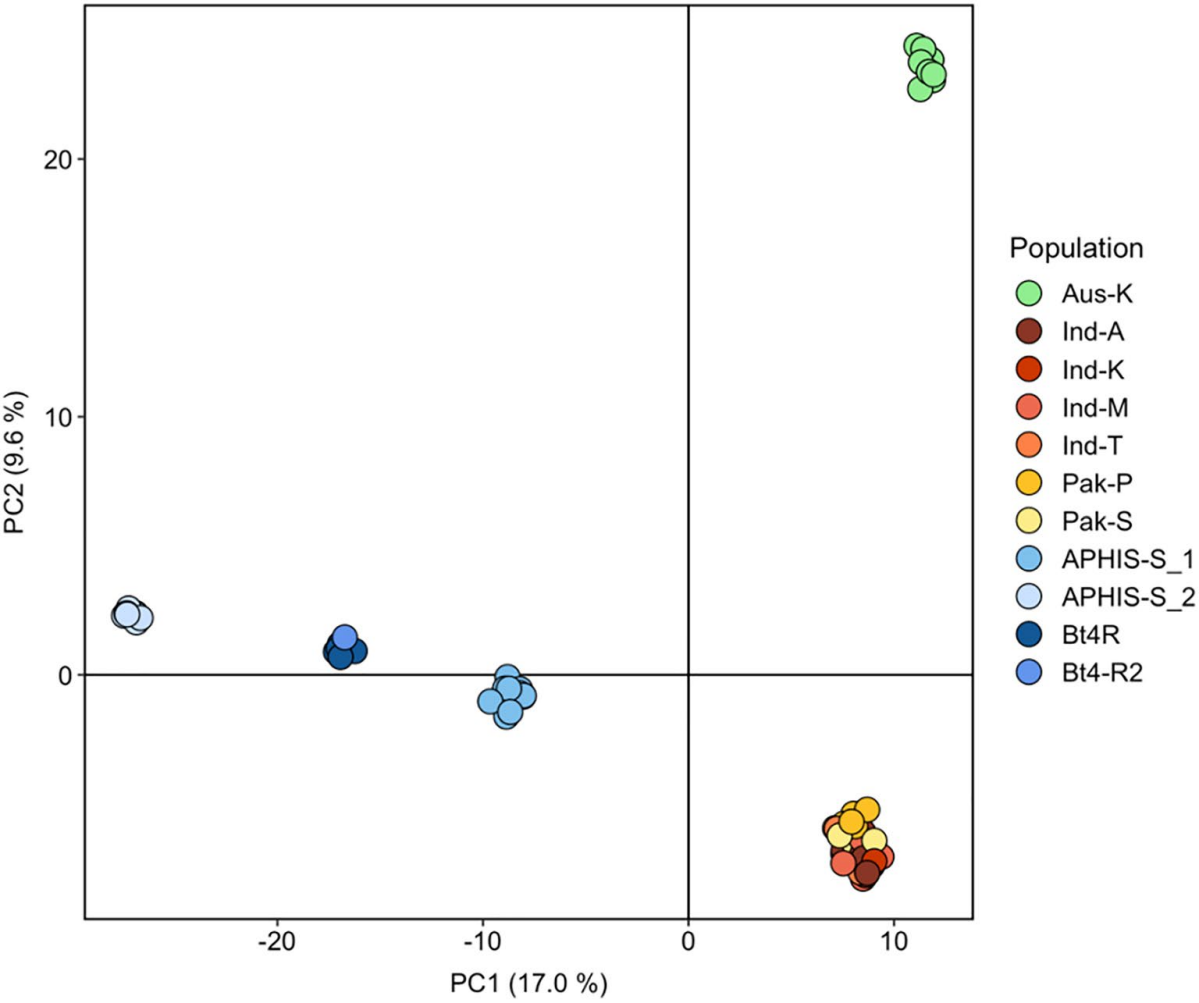
Principal component analysis (PCA) of the filtered dataset of 6355 SNP loci for 11 populations of pink bollworm from Table 1.

**Figure 3:**
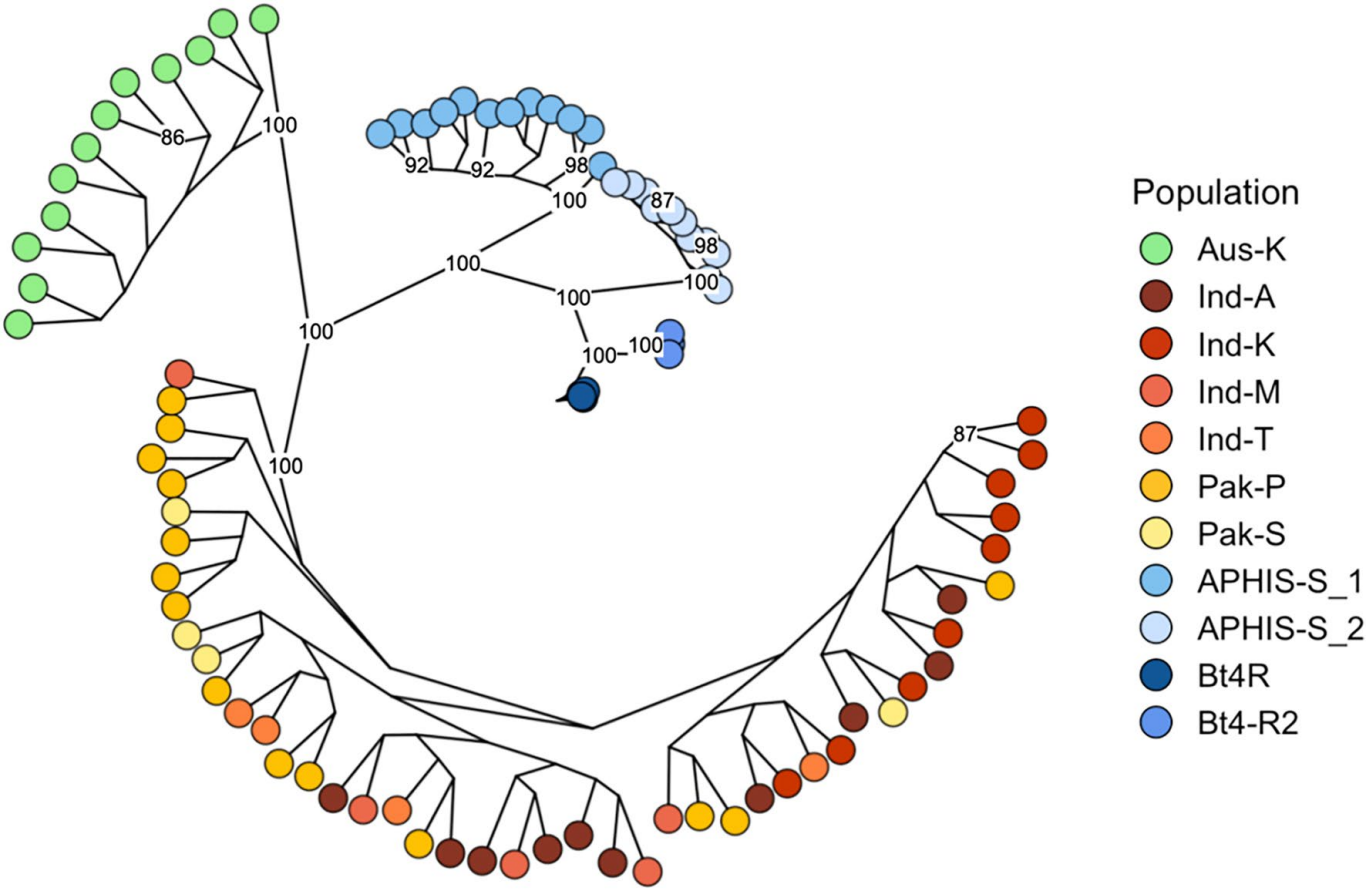
Maximum likelihood tree for individuals from 11 populations of pink bollworm with bootstrap confidence values > 85 attached to nodes.

We observed clear genetic structuring of populations and colonies based on geography, with pairwise F_ST_ values greater than 0.185 for populations pertaining to Australia, U.S., and India-Pakistan. U.S. colonies were strongly genetically differentiated (pairwise F_ST_ = 0.273–0.583; Table 3), whereas Indian and Pakistani populations were genetically similar to one another (pairwise F_ST_ < 0.022; Table 3). These findings were reinforced by the principal component analysis (PCA; Fig. 2) and maximum likelihood (ML) phylogeny (Fig. 3). The first two principal components in the PCA explained 26.6% of the total genetic variance in our dataset, and clearly demonstrated the distinction between individuals assigned to U.S. (blue shades), Australian (green), and India-Pakistan (red/orange/yellow shades) population and colonies (Fig. 2), with a bootstrap confidence value of 100 supporting the divergence of the three main groups in the ML phylogeny (Fig. 3). The PCA assigned U.S. individuals to three main clusters: (1) APHIS-S_1, (2) APHIS-S_2, and (3) Bt4R and Bt4-R2 (Fig. 2). ML further distinguished these groupings, placing each of the four U.S. colonies as genetically distinct with a bootstrap confidence value of 100 (Fig. 3). Consistent with pairwise F_ST_ values, individuals from India and Pakistan lacked population structure, forming one genetic aggregate in the PCA plot and showing limited divergence in the ML phylogeny (Figs. 2, 3).

**Table 3:** Pairwise F_ST_ values for the 11 populations of pink bollworm listed in Table 1.

	Aus-K	Ind-A	Ind-K	Ind-M	Ind-T	Pak-P	Pak-S	APHIS-S_1	APHIS-S_2	Bt4R
Ind-A	0.260	–	–	–	–	–	–	–	–	–
Ind-K	0.259	0	–	–	–	–	–	–	–	–
Ind-M	0.259	0.020	0.014	–	–	–	–	–	–	–
Ind-T	0.255	0.001	0.003	0.007	–	–	–	–	–	–
Pak-P	0.243	0.016	0.011	0.012	0.006	–	–	–	–	–
Pak-S	0.253	0.022	0.021	0.018	0.016	0.003	–	–	–	–
APHIS-S_1	0.347	0.204	0.224	0.206	0.212	0.185	0.211	–	–	–
APHIS-S_2	0.550	0.450	0.549	0.458	0.518	0.408	0.541	0.393	–	–
Bt4R	0.431	0.312	0.369	0.318	0.351	0.287	0.365	0.273	0.558	–
Bt4-R2	0.423	0.310	0.366	0.313	0.337	0.282	0.356	0.327	0.583	0.327

**Figure 4:**
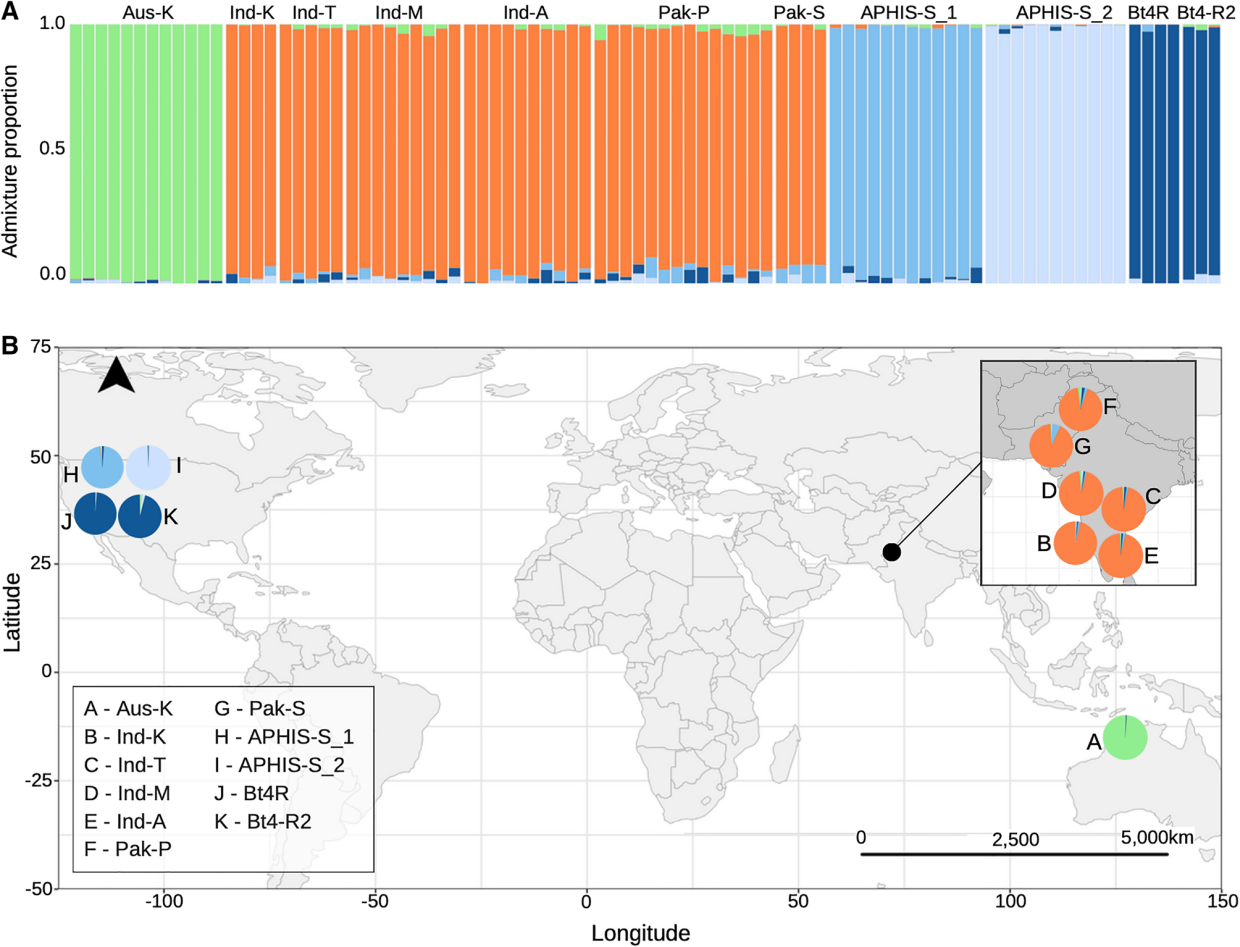
Admixture proportions for 11 populations of pink bollworm from Table 1. (**A**) Each bar represents the admixture coefficient for one individual. (**B**) Geographic distribution of populations with the admixture coefficient for each population represented by a pie chart. Analyses in (**A**) and (**B**) are based on five genetic clusters (K = 5).

Sparse Non-Negative Matrix Factorisation (sNMF) analysis of the filtered dataset (6355 SNPs) indicated an optimal K value of five clusters (Fig. S2; see Fig. S3 for admixture proportions at K = 3, K = 4, and K = 6). These corresponded to Australia, India-Pakistan, and three groups for the U.S. colonies: (1) APHIS-S_2 (light blue), (2) APHIS-S_1 (medium blue), and (3) Bt4R and Bt4-R2 (dark blue) (Fig. 4). We found limited admixture, with each individual corresponding primarily to one genetic group (Fig. 4). The India-Pakistan populations showed the highest absolute admixture, with very small proportions (< ~ 5%) of shared ancestry with U.S. and Australian clusters. Meanwhile, Australia was highly isolated, sharing limited genetic ancestry or migration pathways with other populations (Fig. 4).

**Figure 5:**
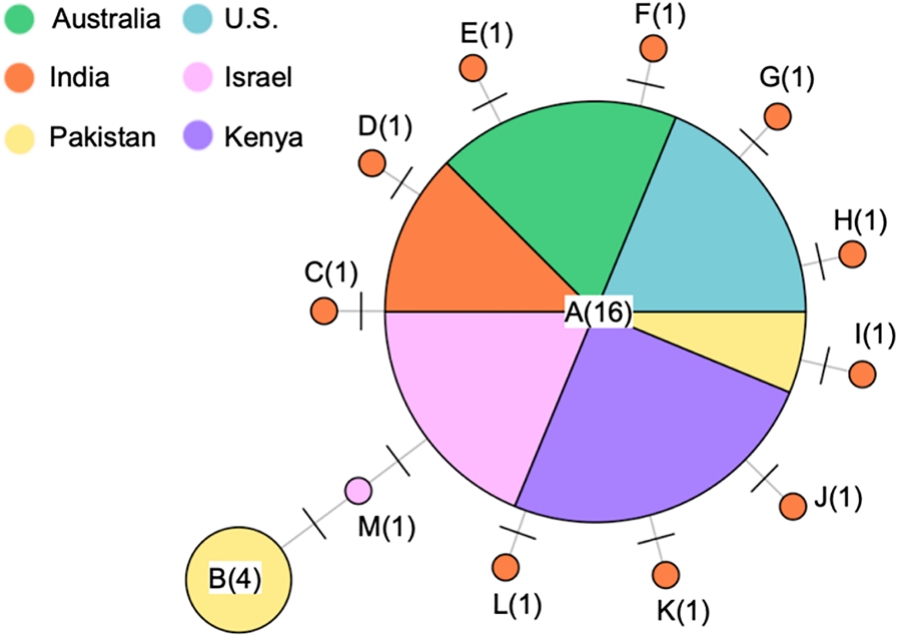
Network of 13 haplotypes (A-M) based on 31 COI sequences from 31 pink bollworm from Australia, India, Pakistan, the U.S., Israel, and Kenya. Circles represent haplotypes, with the circle size proportional to the haplotype frequency and numbers in parentheses indicating the frequency of each haplotype. Lines connecting haplotypes represent mutations, with each dash across a line representing one mutational step between haplotypes.

We identified 13 haplotypes from 31 COI sequences from GenBank, obtained from pink bollworm individuals in six countries: Australia (n = 3 individuals), India (n = 12), Pakistan (n = 5), the U.S. (n = 3), Israel (n = 4), and Kenya (n = 4). Nucleotide diversity was 0.002 in India (10 haplotypes), 0.0007 in Israel (two haplotypes), and 0 in the other four countries (one haplotype each) (Fig. 5). Haplotype A occurred in 16 individuals, including at least one individual from each country (Fig. 5). The second most frequent haplotype (B) occurred in four individuals from Pakistan, but not in any other country (Fig. 5). The other 11 haplotypes (C-M) occurred once each, with 10 from India (C-L) and one from Israel (M). Haplotype B differed by two mutational steps from A, whereas each of the less frequent haplotypes differed by one mutational step from A (Fig. 5).

## Discussion

We used population genomic analyses to assess population structure of seven wild pink bollworm populations from Australia, India, and Pakistan, and four laboratory colonies from the U.S. Overall, our results showed strong population differentiation between continents, including between a population in North West Australia and the other populations and colonies that were sampled in this study. This isolation reduces concerns about the threat of introduction of Bt-resistant insects from India and Pakistan into North West Australia, where the cotton industry is currently expanding.

Conflicting results were obtained in five previous studies using genetic markers to investigate pink bollworm population structure and genetic diversity. For example, using microsatellites^5^, relatively high genetic diversity and clear population structure were found among pink bollworm from China, Pakistan, and the U.S. However, low genetic variation and weak differentiation was later found for the same populations using mtDNA^16^. Among the other studies, high haplotype diversity within populations from Australia, China, India, Israel, Mexico, and the U.S. was found by analysing transposable element sequence data^7^, but low diversity was found in two studies that analysed mtDNA sequences from India^6,17^. These discrepancies may reflect differences between studies in the genetic markers used. Namely, mutation rate likely differs between the marker regions studied, and relative to nuclear DNA, mtDNA is more prone to genetic drift because of its maternal inheritance^16,43,44^. Whereas the previous studies of pink bollworm used one to 13 loci, we analysed genome-wide data for SNPs, which is expected to increase resolution and accuracy for understanding genetic processes^45^.

Our results showed that genetic diversity based on heterozygosity at 6355 SNP markers and nucleotide diversity at mtDNA COI was low within all populations and colonies. As expected, heterozygosity was significantly lower within laboratory colonies from the U.S. compared to within field populations from Australia, India, and Pakistan. However, putative inbreeding (F_IS_) was higher in the field populations. This was surprising as laboratory colonies are expected to show greater F_IS_ because their smaller population sizes increase mating between related individuals^46,47^. However, a meta-analysis of self-incompatible plant species also found that F_IS_ was positively correlated with population size^48^, which is similar to our findings. Estimates of diversity and F_IS_ using SNP data may be influenced by missing data, rare alleles, sample size, and population structure^49^. However, in our study Ho was not correlated with the number of individuals nor the number of field sites sampled per population, and our SNP matrix had < 1.5% missing data. As noted by Schmidt et al.,^49^ more work is needed to evaluate the implications of differences between observed and expected heterozygosity^50^.

Consistent with the microsatellite-based findings of Liu et al.^5^, we found strong genetic differentiation between populations and colonies of pink bollworm from different continents. We also found extremely low admixture between populations and colonies, indicative of low realised gene flow. In the previous studies, strong genetic differentiation, limited gene flow, and low heterozygosity in wild pink bollworm populations was attributed to potential genetic bottlenecks caused by larval mortality from Bt cotton, post-Pleistocene range expansion with limited founders, limited flight activity, and/or narrow host specificity^5–7,16,17^. Pink bollworm moths have been found at altitudes up to 1000 m and their dispersal up to 100 km is documented in the U.S.^51,52^. However, our results imply that inter-continental gene flow is negligible for the populations we studied.

Although pink bollworm is present in over 150 countries worldwide (Fig. 1), genomic data is unavailable for most of these populations. We analysed genomic data from samples obtained at least five to ten years ago from field populations in four to six countries, and four laboratory colonies from the U.S. (see “Methods”). Thus, more recent data from global populations would be valuable. In particular, it would be useful to investigate populations from Indonesia, Phillipines, and Malayasia, as these may represent possible bridgehead intermediaries between India and Australia and may therefore be a potential avenue of spread for Bt-resistant alleles in the future^53^. Future studies might also reconstruct the demographic history of pink bollworm to advance understanding of invasion pathways, and apply selection-based analyses to investigate the evolutionary factors that underpin invasion success (e.g., the specific alleles involved in Bt-resistance)^54^. In such studies, the newly available reference genome for pink bollworm^55^ and wider geographic sampling could yield insights facilitating better monitoring and management of this cosmopolitan pest.

## Supplementary Information

Supplementary Information.

## Data Availability

The data generated and/or analysed during the current study are available in the Figshare digital repository: https://doi.org/10.6084/m9.figshare.22871558.v1.
