# Supplementary material for: Genome-wide analysis reveals distinct global populations of pink bollworm (Pectinophora gossypiella)
Source: Sci Rep. 2023 Jul 20;13:11762. doi: 10.1038/s41598-023-38504-z (PMC10359307; doi:10.1038/s41598-023-38504-z)
Supplement: Supplementary file 1 — Supplementary Information. [file 41598_2023_38504_MOESM1_ESM.pdf]

Genome-wide analysis reveals distinct global populations of pink bollworm (*Pectinophora gossypiella*)

Paige Matheson[1], Elahe Parvizi[1], Jeffrey A. Fabrick[2], Hamid Anees Siddiqui[3,4], Bruce E. Tabashnik[5], Tom Walsh[6], Angela McGaughran[1]

[1]Te Aka Mātuatua - School of Science, University of Waikato, Hamilton, New Zealand

[2] United States Department of Agriculture Agricultural Research Service, United States Arid Land Agricultural Research Center, Maricopa, AZ 85138 USA

[3] Agricultural Biotechnology Division, National Institute for Biotechnology and Genetic Engineering, College Pakistan Institute of Engineering and Applied Sciences (NIBGE-C, PIEAS), Faisalabad, Pakistan.

[4] Synthetic Biology Lab, Department of Biotechnology, University of Sialkot, Sialkot, Pakistan.

[5] Department of Entomology, University of Arizona, Tucson, AZ 85721 USA

[6] Commonwealth Scientific Industrial Research Organisation Environment, Clunies Ross St, Acton, 2601, ACT, Australia

Figure S1. Principal component analysis (PCA) of 11 populations of pink bollworm and one population of spotted pink bollworm (*Pectinophora scutigera*; SPBW). Refer to Table 1 for population code details.

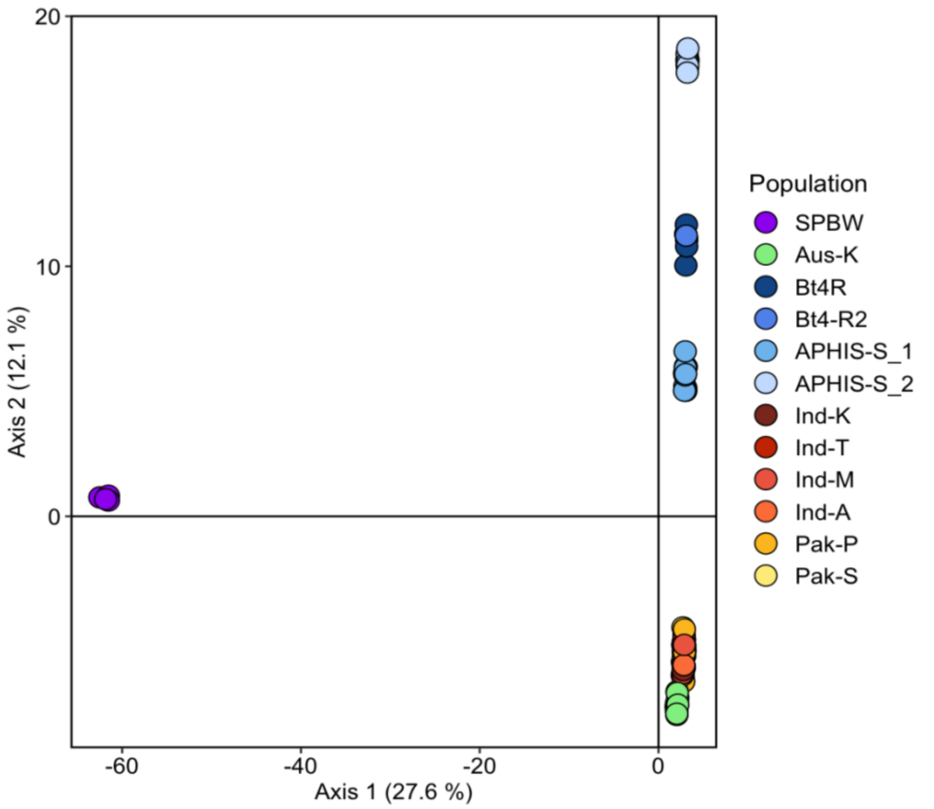

Figure S2. Cross-entropy graph indicating an optimal number of five ancestral populations for K in the admixture (sNMF) analysis.

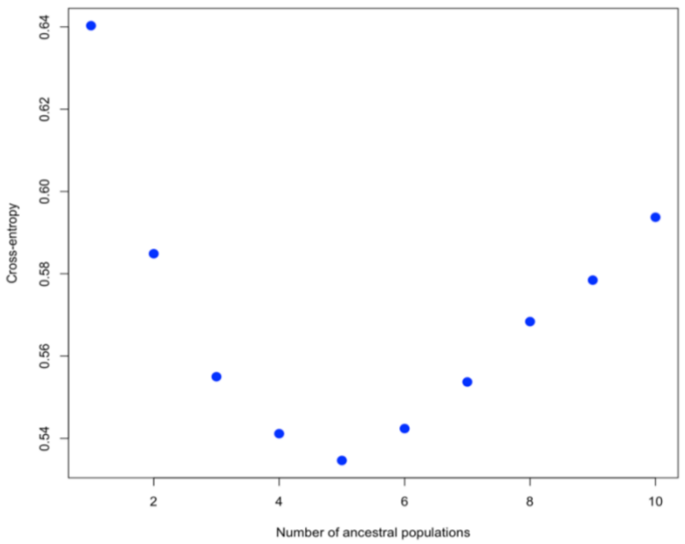

Figure S3. Admixture proportions for 11 populations of pink bollworm. Bar plots represent admixture proportions for each individual at K = 3, K=4, and K=6 as indicated. Refer to Table 1 for population code details.

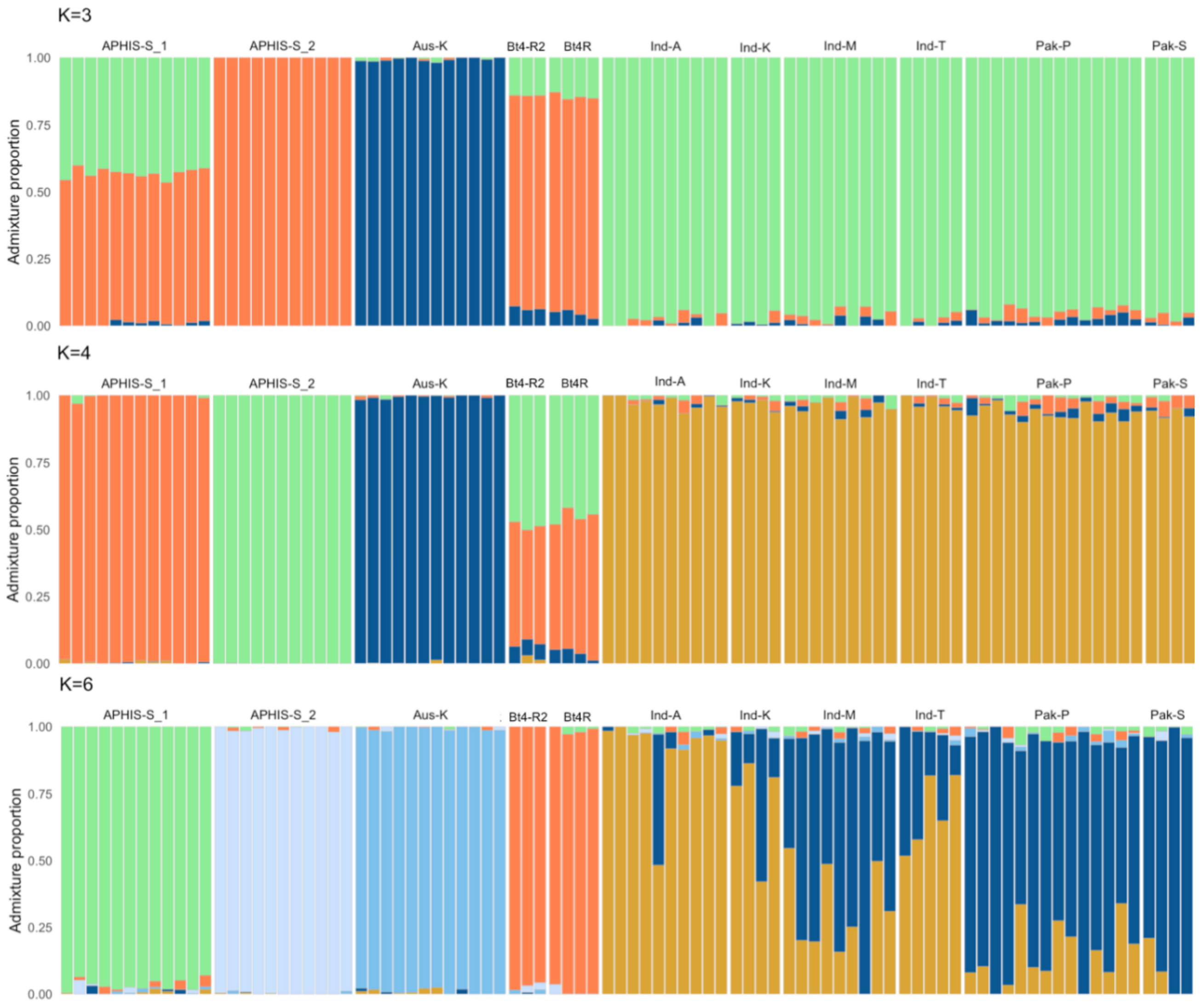

Table S1. Sample information for each pink bollworm individual in the analysis.

Individual	Population	Country	State or Province	City or Lab	Stage
KNX11-4	Aus-K	Australia	Western Australia	Kununurra	Moth (male)
KNX11-5	Aus-K	Australia	Western Australia	Kununurra	Moth (male)
RT1-4	Aus-K	Australia	Western Australia	Kununurra	Moth (male)
RT1-5	Aus-K	Australia	Western Australia	Kununurra	Moth (male)
RT2-1	Aus-K	Australia	Western Australia	Kununurra	Moth (male)
RT2-11	Aus-K	Australia	Western Australia	Kununurra	Moth (male)
RT2-17	Aus-K	Australia	Western Australia	Kununurra	Moth (male)
RT2-3	Aus-K	Australia	Western Australia	Kununurra	Moth (male)
RT2-4	Aus-K	Australia	Western Australia	Kununurra	Moth (male)
RT2-5	Aus-K	Australia	Western Australia	Kununurra	Moth (male)
RT3-2	Aus-K	Australia	Western Australia	Kununurra	Moth (male)
RT3-6	Aus-K	Australia	Western Australia	Kununurra	Moth (male)
ATP-1	Ind-A	India	Andhra Pradesh	Anantapur	Larva
ATP-2	Ind-A	India	Andhra Pradesh	Anantapur	Larva
ATP-3	Ind-A	India	Andhra Pradesh	Anantapur	Larva
ATP-4	Ind-A	India	Andhra Pradesh	Anantapur	Larva
NDL-1	Ind-A	India	Andhra Pradesh	Nandyal	Larva
NDL-2	Ind-A	India	Andhra Pradesh	Nandyal	Larva
P1	Ind-A	India	Andhra Pradesh	Prakasam	Larva
P2	Ind-A	India	Andhra Pradesh	Prakasam	Larva
P3	Ind-A	India	Andhra Pradesh	Prakasam	Larva
P4	Ind-A	India	Andhra Pradesh	Prakasam	Larva
RCR-1	Ind-K	India	Karnataka	Raichur	Larva
RCR-2	Ind-K	India	Karnataka	Raichur	Larva

RCR-3	Ind-K	India	Karnataka	Raichur	Larva
RCR-4	Ind-K	India	Karnataka	Raichur	Larva
MA1	Ind-M	India	Maharashtra	Akola	Larva
MA2	Ind-M	India	Maharashtra	Akola	Larva
MA3	Ind-M	India	Maharashtra	Akola	Larva
MA4	Ind-M	India	Maharashtra	Akola	Larva
MP-1	Ind-M	India	Maharashtra	Parbhani	Larva
W1	Ind-M	India	Maharashtra	Warda	Larva
W2	Ind-M	India	Maharashtra	Warda	Larva
W3	Ind-M	India	Maharashtra	Warda	Larva
W4	Ind-M	India	Maharashtra	Warda	Larva
KNL-2	Ind-T	India	Telangana	Karnool	Larva
KNL-3	Ind-T	India	Telangana	Karnool	Larva
WGL-1	Ind-T	India	Telangana	Warangal	Larva
WGL-2	Ind-T	India	Telangana	Warangal	Larva
WGL-4	Ind-T	India	Telangana	Warangal	Larva
Pk1.1	Pak-P	Pakistan	Punjab	Faisalabad	Larva
Pk1.2	Pak-P	Pakistan	Punjab	Faisalabad	Larva
Pk1.3	Pak-P	Pakistan	Punjab	Faisalabad	Larva
Pk1.4	Pak-P	Pakistan	Punjab	Faisalabad	Larva
Pk10.1	Pak-P	Pakistan	Punjab	Bahawalpur	Larva
Pk10.2	Pak-P	Pakistan	Punjab	Bahawalpur	Larva
Pk2.1	Pak-P	Pakistan	Punjab	Toba Tek Singh	Larva
Pk3.1	Pak-P	Pakistan	Punjab	Toba Tek Singh	Larva
Pk4.1	Pak-P	Pakistan	Punjab	Multan	Larva
Pk4.2	Pak-P	Pakistan	Punjab	Multan	Larva

Pk5.1	Pak-P	Pakistan	Punjab	Multan	Larva
Pk5.2	Pak-P	Pakistan	Punjab	Multan	Larva
Pk7.1	Pak-P	Pakistan	Pubjab	Vehari	Larva
Pk8.1	Pak-P	Pakistan	Pubjab	Vehari	Larva
Pk18.1	Pak-S	Pakistan	Sindh	Hyderabad	Larva
Pk18.2	Pak-S	Pakistan	Sindh	Hyderabad	Larva
Pk18.3	Pak-S	Pakistan	Sindh	Hyderabad	Larva
Pk18.4	Pak-S	Pakistan	Sindh	Hyderabad	Larva
APHIS-1	APHIS-S_1	U.S.	Arizona	Lab	4th instar larva
APHIS-18	APHIS-S_1	U.S.	Arizona	Lab	4th instar larva
APHIS-19	APHIS-S_1	U.S.	Arizona	Lab	4th instar larva
APHIS-2	APHIS-S_1	U.S.	Arizona	Lab	4th instar larva
APHIS-20	APHIS-S_1	U.S.	Arizona	Lab	4th instar larva
APHIS-21	APHIS-S_1	U.S.	Arizona	Lab	4th instar larva
APHIS-22	APHIS-S_1	U.S.	Arizona	Lab	4th instar larva
APHIS-23	APHIS-S_1	U.S.	Arizona	Lab	4th instar larva
APHIS-3	APHIS-S_1	U.S.	Arizona	Lab	4th instar larva
APHIS-4	APHIS-S_1	U.S.	Arizona	Lab	4th instar larva
APHIS-5	APHIS-S_1	U.S.	Arizona	Lab	4th instar larva
APHIS-6	APHIS-S_1	U.S.	Arizona	Lab	4th instar larva
APHIS-41	APHIS-S_2	U.S.	Arizona	Lab	4th instar larva
APHIS-42	APHIS-S_2	U.S.	Arizona	Lab	4th instar larva
APHIS-43	APHIS-S_2	U.S.	Arizona	Lab	4th instar larva
APHIS-44	APHIS-S_2	U.S.	Arizona	Lab	4th instar larva
APHIS-45	APHIS-S_2	U.S.	Arizona	Lab	4th instar larva
APHIS-46	APHIS-S_2	U.S.	Arizona	Lab	4th instar larva

APHIS-53	APHIS-S_2	U.S.	Arizona	Lab	4th instar larva
APHIS-54	APHIS-S_2	U.S.	Arizona	Lab	4th instar larva
APHIS-55	APHIS-S_2	U.S.	Arizona	Lab	4th instar larva
APHIS-56	APHIS-S_2	U.S.	Arizona	Lab	4th instar larva
APHIS-57	APHIS-S_2	U.S.	Arizona	Lab	4th instar larva
Bt4R-6	Bt4R	U.S	Arizona	Lab	4th instar larva
Bt4R-7	Bt4R	U.S	Arizona	Lab	4th instar larva
Bt4R-8	Bt4R	U.S	Arizona	Lab	4th instar larva
Bt4R-9	Bt4R	U.S	Arizona	Lab	4th instar larva
Bt4R2-6	Bt4-R2	U.S	Arizona	Lab	4th instar larva
Bt4R2-7	Bt4-R2	U.S	Arizona	Lab	4th instar larva
Bt4R2-8	Bt4-R2	U.S	Arizona	Lab	4th instar larva

Table S2. Sample information for COI sequences downloaded from NCBI database (https://www.ncbi.nlm.nih.gov/).

**Sample**	**Accession number**	**Genotype**	**Location information**	**Author**
US_1	JF815079	A	University of Arizona, U.S.	Moore & Hughes, 2011
US_2	JF815080	A	University of Arizona, U.S.	Moore & Hughes, 2011
US_3	JF815081	A	University of Arizona, U.S.	Moore & Hughes, 2011
Aus_1	KF387796	A	Western Australia	McKeown et al. 2013
Aus_2	KF391287	A	Kununurra, Western Australia	Hebert et al. 2013
Aus_3	KF394480	A	Gulon Point, Northern Territory, Australia	Hebert et al. 2013
India_1	KM289071	A	Shankarnagar, Maharashtra, India	Sridhar et al. 2014
India_2	KM289072	A	Shankarnagar, Maharashtra, India	Sridhar et al. 2014
India_3	KM289073	C	Shankarnagar, Maharashtra, India	Sridhar et al. 2014
India_4	KM289074	D	Shankarnagar, Maharashtra, India	Sridhar et al. 2014
India_5	KM289075	E	Shankarnagar, Maharashtra, India	Sridhar et al. 2014
India_6	KM289076	F	Shankarnagar, Maharashtra, India	Sridhar et al. 2014
India_7	KM289077	G	Shankarnagar, Maharashtra, India	Sridhar et al. 2014
India_8	KM289078	H	Shankarnagar, Maharashtra, India	Sridhar et al. 2014
India_9	KM289079	I	Shankarnagar, Maharashtra, India	Sridhar et al. 2014
India_10	KM289080	J	Shankarnagar, Maharashtra, India	Sridhar et al. 2014
India_11	KM289081	K	Shankarnagar, Maharashtra, India	Sridhar et al. 2014
India_12	KM289082	L	Shankarnagar, Maharashtra, India	Sridhar et al. 2014
Pakistan_1	KX860287	A	Faisalabad, Punjab, Pakistan	Ashfaq et al. 2017
Pakistan_2	KX861047	B	Faisalabad, Punjab, Pakistan	Ashfaq et al. 2017
Pakistan_3	KX862450	B	Faisalabad, Punjab, Pakistan	Ashfaq et al. 2017
Pakistan_4	KX862806	B	Faisalabad, Punjab, Pakistan	Ashfaq et al. 2017
Pakistan_5	KX863147	B	Faisalabad, Punjab, Pakistan	Ashfaq et al. 2017

Kenya_1	MF121861	A	Siaya, Rarieda, Obaga, Kenya	Kinyanjui et al. 2017
Kenya_2	MF121862	A	Siaya, Rarieda, Obaga, Kenya	Kinyanjui et al. 2017
Kenya_3	MF121863	A	Siaya, Rarieda, Obaga, Kenya	Kinyanjui et al. 2017
Kenya_4	MF121864	A	Siaya, Rarieda, Obaga, Kenya	Kinyanjui et al. 2017
Israel_1	JF815075	M	Israel	Moore & Hughes, 2011
Israel_2	JF815076	A	Israel	Moore & Hughes, 2011
Israel_3	JF815078	A	Israel	Moore & Hughes, 2011
Israel_4	JF815077	A	Israel	Moore & Hughes, 2011
